# Identification of a Disulfide Bridge in Sodium-Coupled Neutral Amino Acid Transporter 2(SNAT2) by Chemical Modification

**DOI:** 10.1371/journal.pone.0158319

**Published:** 2016-06-29

**Authors:** Chen Chen, Jiahong Wang, Ruiping Cai, Yanmeng Yuan, Zhanyun Guo, Christof Grewer, Zhou Zhang

**Affiliations:** 1 College of Life Science and Biopharmacy, Shenyang Pharmaceutical University, Shenyang 110015, People’s Republic of China; 2 Institute of Protein Research, College of Life Sciences and Technology, Tongji University, Shanghai 200092, People’s Republic of China; 3 Departments of Chemistry and Biological Sciences, Binghamton University, Binghamton, New York, 13902, United States of America; University of Cambridge, UNITED KINGDOM

## Abstract

Sodium-coupled neutral amino acid transporter 2 (SNAT2) belongs to solute carrier 38 (SLC38) family of transporters, which is ubiquitously expressed in mammalian tissues and mediates transport of small, neutral amino acids, exemplified by alanine(Ala, A). Yet structural data on SNAT2, including the relevance of intrinsic cysteine residues on structure and function, is scarce, in spite of its essential roles in many tissues. To better define the potential of intrinsic cysteines to form disulfide bonds in SNAT2, mutagenesis experiments and thiol-specific chemical modifications by N-ethylmaleimide (NEM) and methoxy-polyethylene glycol maleimide (mPEG-Mal, MW 5000) were performed, with or without the reducing regent dithiothreitol (DTT) treatment. Seven single mutant transporters with various cysteine (Cys, C) to alanine (Ala, A) substitutions, and a C245,279A double mutant were introduced to SNAT2 with a hemagglutinin (HA) tag at the C-terminus. The results showed that the cells expressing C245A or C279A were labeled by one equivalent of mPEG-Mal in the presence of DTT, while wild-type or all the other single Cys to Ala mutants were modified by two equivalents of mPEG-Mal. Furthermore, the molecular weight of C245,279A was not changed in the presence or absence of DTT treatment. The results suggest a disulfide bond between Cys245 and Cys279 in SNAT2 which has no effect on cell surface trafficking, as well as transporter function. The proposed disulfide bond may be important to delineate proximity in the extracellular domain of SNAT2 and related proteins.

## Introduction

The SLC38 family of transporters represents a main branch of solute carrier families in mammals [1]. Most of the 11 transporters in this family are Na^+^-dependent and are able to carry out net transport of neutral amino acids except 5 orphan transporters [2]. The other six of these family members have been well-characterized to date and have been subdivided into System A (SNAT1, SNAT2 and SNAT4) [3–6].and System N (SNAT3, SNAT5 and SNAT7) [7–11] transporters, in terms of their functional properties and patterns of regulation. System A prefers small aliphatic amino acids, while System N has a much narrower substrate profiles of glutamine, asparagine and histidine [12, 13]. In addition, System N has the ability to co-transport Na^+^ and antiport H^+^ [5,14]. However, System A transports substrates coupled to the uptake of Na^+^ with a stoichiometry of 1:1 [15].

Sodium-coupled neutral amino acid transporter 2 (SNAT2, previously ATA2, SA1, or SAT2) is a prototype for System A which has a very broad tissue distribution profile [16–22]. SNAT2 mediates transportation of a broad range of zwitterionic, aliphatic amino acids, such as alanine, asparagine, cysteine, glutamine, glycine, histidine, methionine, proline, and serine. SNAT2 operates by a mechanism with ordered binding (Na^+^ first) and 1 Na^+^: 1 amino acid coupling stoichiometry. It has been reported that SNAT2 is assigned many important physiological roles. SNAT2 has been suggested to be involved in glutamine transport in the glutamate/glutamine cycle in neurons with some other members of SLC38 family [13, 23–26]. In the liver, SNAT2 plays a significant role in the gluconeogenesis and ammonia detoxification from portal blood [27, 28]. The increasing expression of SNAT2 in hypertonically stressed cells indicates the role of SNAT2 in the cell volume regulation [29–31]. Furthermore, SNAT2 appears to play a role in cell growth and differentiation by signaling through the mTOR pathway [32, 33]. There is evidence that SNAT2 is up-regulated in cancer, for instance, human liver cancer and prostate cancer [34].

In contrast to our broad knowledge of SNAT2’s functional roles, there is relatively little known about its structure. SNAT2 contains 504 amino acids residues with a predicted molecular mass of 56 kDa. Hydropathy plotting indicates that SNAT2 has 11 transmembrane helices (TM) with an intracellular N terminus and an extracellular C terminus [1, 20]. Homology models of SNAT2 based on the structure of LeuT_Aa_/Mhp1 show a tandem duplication between segments containing TM 1–5 and TM 6–10 [35]. Previously, we have shown that Histidine (His) 304 is required for the transport of Ala [36]. Asparagine (Asn) 82 in TM1 and Threonine (Thr) 384 in TM8 are proposed to form a possible Na^+^ binding site [35, 37]. Furthermore, a large portion of the C-terminus of SNAT2 plays a significant role for amino acid translocation and its voltage dependence [38]. The highly conserved C-terminus His504 is reported to be related to the pH-sensitivity of SNAT2 [39].

Disulfide bridges between correct pairs of cysteine residues are crucial to the trafficking [40], stability [41, 42] and function [43–46] of transmembrane proteins. A recent study has shown that the disulfide bond formed by Cys249 and Cys321 plays an essential role in the substrate transport of SNAT4 [47], another member of System A, which shares 57% sequence homology with SNAT2. Better understanding of potential disulfide bridge formation of SNAT2 is vital to the delineation of its transport mechanism. Because of the limited expression and purification of SNAT2 protein, it is difficult to use conventional methods that are only feasible with proteins in abundant quantities, such as crystallization and NMR. In this study, therefore, we employed a combination of mutagenesis and thiol-specific chemical modifications by N-ethylmaleimide (NEM) and methoxy-polyethylene glycol maleimide (mPEG-Mal, MW 5000).

Here, we first prepared extracts from HEK 293T cells that express wild-type SNAT2 and performed thiol-specific chemical modification by sequential treatment with NEM, DTT, and mPEG-Mal under denatured condition. After assessing the influence of DTT on the molecular weight of the wild-type transporter, systematic mutagenesis was performed with various single Cys replaced with Ala, and these mutant transporters were modified with the same method as wild-type SNAT2. Immunofluorescence methods were used to detect the expression levels and localization of the mutants. The result suggested that a disulfide bridge is formed between Cys245 and Cys279 which, however, has little effect on the trafficking to cell surface of SNAT2, as well as transport function of SNAT2.

## Materials and Methods

### Materials

PEG-Mal was purchased from Laysan Bio. NEM was from Sigma. DTT was bought from Biosharp. Oligonucleotide primers were synthetized by SanGon. La Taq DNA polymerase was from TaKaRa. Human Embryo Kidney cells (HEK293T, ATCC number CRL 11268) were purchased from the Typical Culture Collection cell bank, Chinese Academy of Sciences. Dulbecco’s modified Eagle’s medium (DMEM) was bought from Gibco. Lipofectamine 2000 transfection reagent and Alexa Fluor 568 donkey anti-mouse IgG secondary antibody were purchased in Invitrogen. PVDF membrane and Chemiluminescence HRP Substrate were acquired from Millipore. Anti-HA tag mouse monoclonal antibody and Anti-GAPDH mouse monoclonal antibody were purchased from ImB. HRP-linked goat anti-mouse IgG secondary antibody and protein inhibitors were bought from Sigma. Polaroid films were bought from Kodak.

### Site-directed mutagenesis of SNAT2

Mutants of SNAT2 were constructed using the rat SNAT2 with an HA tag at the C-terminus in a eukaryotic expression vector p*BK-CMVΔ(1098–1300)* (p*BK-CMVΔ-SNAT2- HA*). Point mutations were introduced using the primers listed in Table 1. Cysteine residues at position 228, 238, 245, 279, 303, 401, and 475 were mutated to alanine or serine, respectively. Double mutation was generated by mutating both Cys245 and Cys279 to alanine or serine. In all mutants, introduction of the correct mutation were confirmed by DNA sequencing.

**Table 1:** DNA primers used for site-directed mutagenesis.

Mutation sites	Sense	Antisense
**C228A**	**CAGCGGCCTTTCTCT****GCT****C****GCT****ATGATATTCTTTCTGATTG**	**CAATCAGAAAGAATATCAT****AGC****GAGCAGAGAAAGGCCGCTG**
**C238A**	**CTTTCTGATTGTGGTGATT****GCC****AAGAAGTTTCAGATTCC**	**GGAATCTGAAACTTCTT****GGC****AATCACCACAATCAGAAAG**
**C245A**	**GAAGTTTCAGATTCCT****GCC****CCTGTGGAAGTGGC**	**GCCACTTCCACAGG****GGC****AGGAATCTGAAACTTC**
**C279A**	**CAGCCGATACC****GCC****AGGCCCCGTTACTTTATC**	**GATAAAGTAACGGGGCCT****GGC****GGTATCGGCTG**
**C303A**	**GACGTTTTCCTTTGTC****GCC****CATCCCGCTGTCCTTC**	**GAAGGACAGCGGGATG****GGC****GACAAAGGAAAACGTC**
**C401A**	**CGGTCACTCACTTACTG****GCC****CCTACAAAAGAGTTCAG**	**CTGAACTCTTTTGTAGG****GGC****CAGTAAGTGAGTGACCG**
**C475A**	**CAAAAGATTGGGGCTCTG****GCT****TTTCTCCTGAGTGGCG**	**CGCCACTCAGGAGAAA****AGC****CAGAGCCCCAATCTTTTG**
**C228S**	**CAGCGGCCTTTCTCTGCT****CTC****TATGATATTCTTTCTGATTG**	**CAATCAGAAAGAATATCAT****AGA****GAGCAGAGAAAGGCCGCTG**
**C238S**	**CTTTCTGATTGTGGTGATT****TCC****AAGAAGTTTCAGATTCC**	**GGAATCTGAAACTTCTT****GGA****AATCACCACAATCAGAAAG**
**C245S**	**GAAGTTTCAGATTCCT****AGC****CCTGTGGAAGTGGC**	**GCCACTTCCACAGG****GCT****AGGAATCTGAAACTTC**
**C279S**	**CAGCCGATACC****TCC****AGGCCCCGTTACTTTATC**	**GATAAAGTAACGGGGCCT****GGA****GGTATCGGCTG**
**C303S**	**GACGTTTTCCTTTGTC****TCC****CATCCCGCTGTCCTTC**	**GAAGGACAGCGGGATG****GGA****GACAAAGGAAAACGTC**
**C401S**	**CGGTCACTCACTTACTG****TCC****CCTACAAAAGAGTTCAG**	**CTGAACTCTTTTGTAGG****GGA****CAGTAAGTGAGTGACCG**
**C475S**	**CAAAAGATTGGGGCTCTG****TCT****TTTCTCCTGAGTGGCG**	**CGCCACTCAGGAGAAA****AGA****CAGAGCCCCAATCTTTTG**

### Cell culture and transfection

HEK293T cells were cultured in Dulbecco’s modified Eagle’s medium (DMEM), supplemented with 10% FBS, 4 mM L-glutamine, 100 U/ml penicillin and 100 μg/ml streptomycin at 37°C in a humidified incubator under an atmosphere of 5% CO_2_ in air. After plating the cells on 35-mm dishes for 24 h, plasmids of either p*BK-CMVΔ-SNAT2-HA* wild type or mutant transporter were transiently transfected into cells respectively using Lipofectamine 2000 according to the manufacturer’s protocol.

### Extraction of cell total proteins

After transfection for 24 h, HEK293T cells were washed 2–3 times with ice-cold PBS containing 0.1 mM CaCl_2_, 1mM MgCl_2_ and then lysed in RIPA lysis buffer containing 100 mM Tris-Cl (pH 7.4), 150 mM NaCl, 1mM EDTA, 1% Triton X-100, 1% sodium deoxycholate, 0.1% SDS and protease inhibitor. Freeze-thawing technique was used to break the cells. The intact cells and nucleus were removed by centrifugation at 1000 g for 15 min at 4°C. The supernatant was removed and represents the total protein fraction. Protein concentrations were determined with BCA protein assay kit according to the manufacturer’s instruction.

### Immunofluorescence microscopy

Immunofluorescence staining was performed on transiently transfected HEK293T cells grown on glass coverslips in 35-mm dishes 24 h after transfection. Cells were washed with phosphate-buffered saline (PBS) for 3 times, and then fixed with 4% paraformaldehyde in PBS for 30 min. After washed twice with PBS to remove the excess of paraformaldehyde, the cells were incubated with 1% bovine serum albumin (BSA) in PBS to block the nonspecific binding. Cells were then incubated with anti-HA tag mouse monoclonal antibody (1:1000 dilution) and Alexa Fluor 568 donkey anti-mouse IgG (1:500 dilution) in the dark for 2 h in sequence. Nuclei were stained with 4,6-diamidino-2-phenylindole dihydrochloride (DAPI; 1:1000 dilution). All steps were performed at room temperature. Images were obtained by using a Leica TCS SP5 confocal laser-scanning microscope.

### Protein modification with NEM and mPEG-Mal

NEM, DTT, and mPEG-Mal solution were freshly prepared in lysate buffer (10% SDS, 1 mM EDTA, 50 mM Tris-HCl, pH 7.5). After transfection for 24 h, HEK293T cells cultured in 3.5mm dishes were washed 2–3 times with PBS and scraped into 100 μL of lysate buffer supplemented with 50mM NEM or DTT. The modification reaction was carried out for 30 min. Excess NEM or DTT in the lysate were removed by chloroform/methanol precipitation method with methanol/chloroform/water (4:1:3 V/V/V) treatment in order. Subsequently, the experimental group and control group pellets were respectively dissolved in the lysate buffer with or without 50 mM DTT or 50 mM NEM, and then incubated for 30 min. After precipitated by methanol/chloroform/water as described above, the proteins were dissolved in the lysate Buffer containing 8 mM mPEG-Mal and incubated for 30 min. All these modification steps were performed at room temperature.

### SDS-PAGE and Western blotting

Cell lysates and modified proteins were fractionated by 8% SDS-PAGE and electrotransferred onto a PVDF membrane. Nonspecific binding was blocked with TBST containing 0.1% Tween 20 and 5% skim milk. The membrane was then incubated for 3 h with the primary antibody (anti-HA tag and anti-GAPDH mouse monoclonal antibody) diluted 1:3000 in 2.5% skim milk in TBST. The secondary antibody (HRP-conjugated goat anti-mouse IgG antibody) was used in a dilution of 1:10000 in 2.5% skim milk in TBST for 1 h at room temperature. Bands were visualized using an enhanced chemiluminescence detection method with ECL Western blotting detection kit. The bands are smeared because there are two predicted glycosylation sites in the SNAT2.

### Amino acid uptake assay

HEK293T cells were plated on collagen-coated 12-well plates (1×10^5^/well) in DMEM (containing 10% fetal bovine serum, 100 units/mL of penicillin, 10 μg/mL of streptomycin, and 4 mM of glutamine. 48h after transfection with vector, wild-type SNAT2, or SNAT2 serine mutants cDNA, the cells were washed with uptake buffer (containing 140 mM sodium methaneuslfonate, 2 mM magnesium methanesulfonate, 2 mM calcium gluconate, 30 mM Tris-Mes, pH 8.0, 5 mM glucose) 2 times. The cells were preincubated in the same buffer for 5 min at 37°C, then the buffer was removed and replaced with fresh buffer containing unlabeled α-methyl-amino-isobutyric acid (MeAIB) and 0.4 μCi of [^14^C] MeAIB (PerkinElmer Life Sciences; total concentration 40 μM). Uptake was terminated by washing twice with 1 mL of uptake buffer on ice after 1 min of incubation at room temperature. The cells were then solubilized in 0.5 mL of 1% SDS, and radioactivity was measured by scintillation couting in 3 ml of scintillation fluid. The MeAIB uptake measurements were performed in triplicate.

### Whole-cell current recording

HEK293T cells cultures were transiently transfected with wild-type or mutant cDNA. One day after transfection, the cells were used for electrophysiological measurements. Alanine-induced SNAT2 currents were recorded in the whole-cell current recording configuration. Whole-cell currents were recorded with an EPC7 patch clamp amplifier (ALA Scentific, Westbury, NY) at steady state. The resistance of the recording was 2–3 megohms, as described previously [35–37].

### Statistical analysis

Data shown are presented as the mean ± standard deviation (SD). Differences between 2 groups were examined for statistical significance using the Student’s t-test. When more than two groups were compared, analysis of Variance (ANOVA) is used to determine statistical significance.

## Results

### Identifying a disulfide bridge in wild-type SNAT2-HA

**Fig 1:**
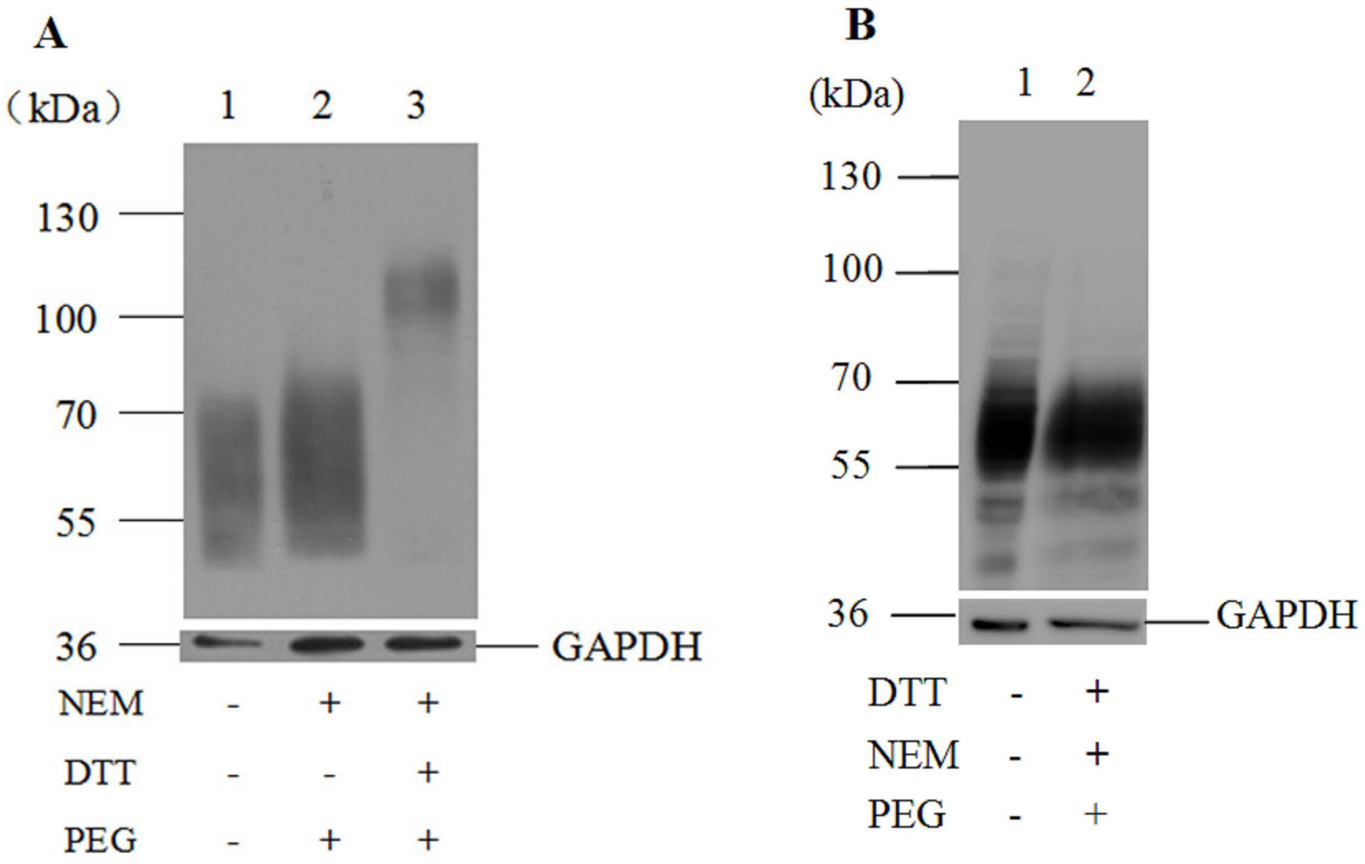
Western blotting analysis of wild-type SNAT2-HA modified by NEM and mPEG-Mal. Lysates of HEK293T cells expressing wild-type SNAT2-HA were processed by sequential treatment of NEM, DTT, and mPEG-Mal (A) or DTT, NEM, and mPEG-Mal (B) under denatured condition. Controls were treated without DTT. The proteins were separated on an 8% SDS gel, detected by immunodetection with the mouse anti-HA tag antibody followed by the HRP-conjugated goat anti-mouse secondary antibody. Anti-GAPDH mouse monoclonal antibody was used to detect the GAPDH as an internal reference.

In order to identify the existence of potential disulfide bridges in SNAT2, a chemical modification approach was performed utilizing wild-type SNAT2-HA with or without the reducing reagent DTT. Thiol-specific reagents NEM and mPEG-Mal were employed, both of which can only react with free sulfhydryls in proteins. NEM has a small molecular weight of 125 Da, which has little effect on the mobility of covalent binding proteins in polyacrylamide gels, as detected by Western blotting analysis. In comparison, modification by mPEG-Mal can be easily detected on SDS-PAGE. By virtue of the hydrodynamic properties of mPEG-Mal, the PEG adducts run with a slower mobility on SDS-PAGE. Adding one PEG molecule will increase the apparent molecular mass of the parent protein by 10∼15 kDa, in spite of the fact that PEG carbohydrate moiety is only 5 kDa. As can be seen in Fig 1A (lane 2), after modification by NEM, the migration rate of the modified wild-type SNAT2-HA was consistent with that of the unmodified SNAT2-HA on SDS-PAGE. After blocking all of the free thiols with NEM and followed by DTT treatment under denaturing conditions, a SNAT2-HA band with a larger molecular mass of about 110 kDa could be observed (Fig 1A, lane 3), suggesting that cysteine residues of SNAT2 may be engaged in disulfide bridges. In contrast, when the wild-type SNAT2-HA was treated with DTT firstly, and subsequently modified by NEM, followed by PEG, the migration rate was consistent with that of unmodified SNAT2-HA (Fig 1B, lane 1 and 2). These results indicate that SNAT2 HA can be modified by PEG when thiols forming a potential disulfide bridge are reduced through DTT treatment, but only before they are blocked from PEG modification by NEM.

### Residues Cys245 and Cys279 are linked by a disulfide bridge

To identify the cysteine residues involved in the formation of the disulfide bond, single mutants were constructed by substituting each of the seven cysteine residues in SNAT2-HA with alanine. All of these mutant transporters were modified sequentially by NEM and mPEG-Mal, in the presence or absence of DTT.

**Fig 2:**
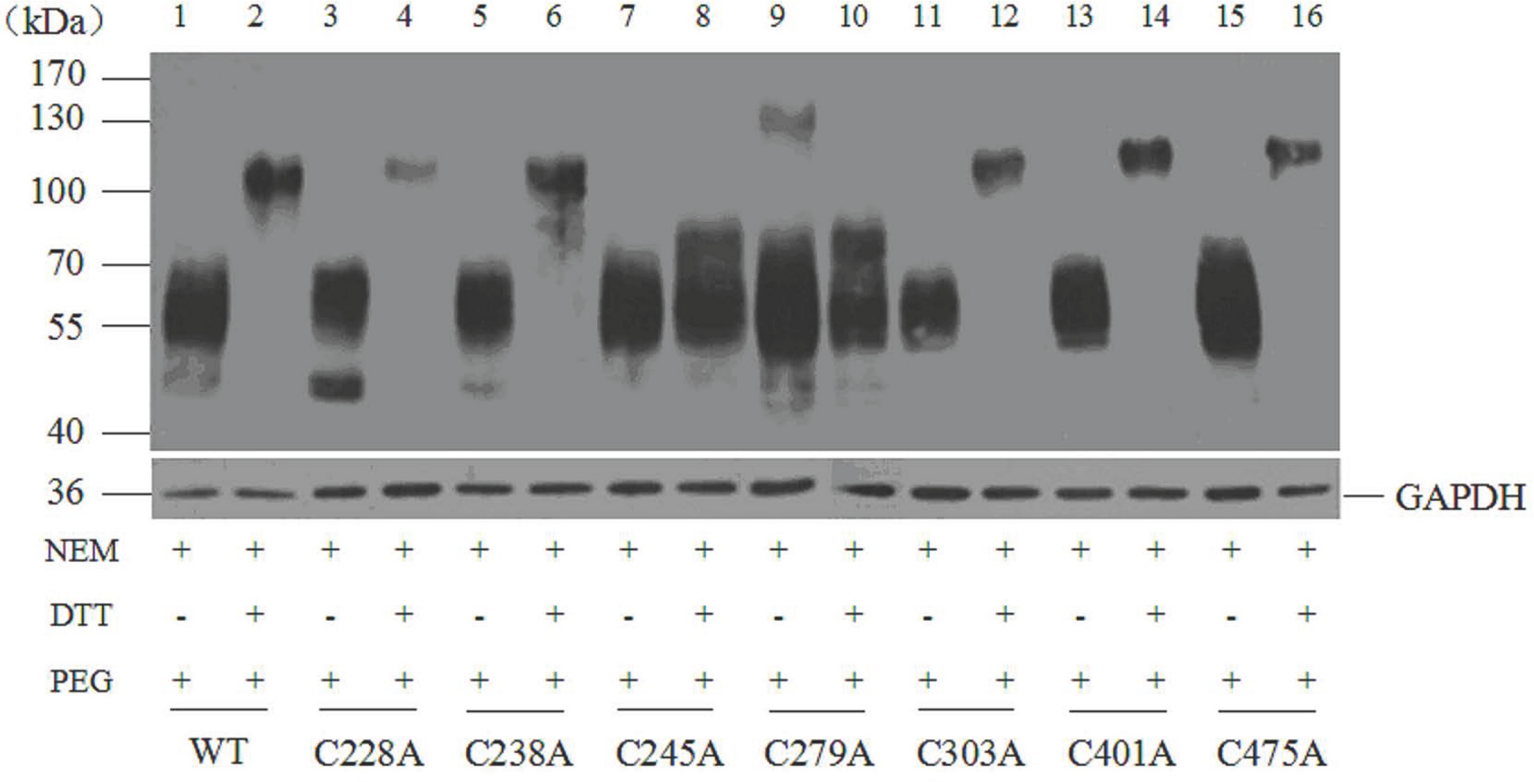
Western blotting analysis of SNAT2-HA wild-type and 7 single cysteine mutants modified by NEM and mPEG-Mal implicates Cys245 and Cys279 in a disulfide bridge. Lysates of HEK293T cells expressing either wild-type or 7 single cysteine mutants were processed by sequential treatment of NEM, DTT, and mPEG-Mal under denatured condition. Controls were treated without DTT. The proteins were separated on an 8% SDS gel, detected by immunodetection with the mouse anti-HA tag antibody followed by the HRP-conjugated goat anti-mouse secondary antibody. Anti-GAPDH mouse monoclonal antibody was used to detect the GAPDH as an internal reference.

For the mutant transporters C228A, C238A, C303A, C401A, and C475A, the protein apparent molecular weight was shifted from 55~70 kDa in the absence of DTT (Fig 2, lane 3, 5, 11, 13, and 15) to 110 kDa after DTT treatment (Fig 2, lane 4, 6, 12, 14, and 16), consistent with that of the wild-type transporter (Fig 2, lane 1 and 2). The result indicated the existence of disulfide bond in these mutant transporters, which means residues Cys 228, 238, 303, 401, and 475 did not participate in the formation of disulfide bond of SNAT2.

**Fig 3:**
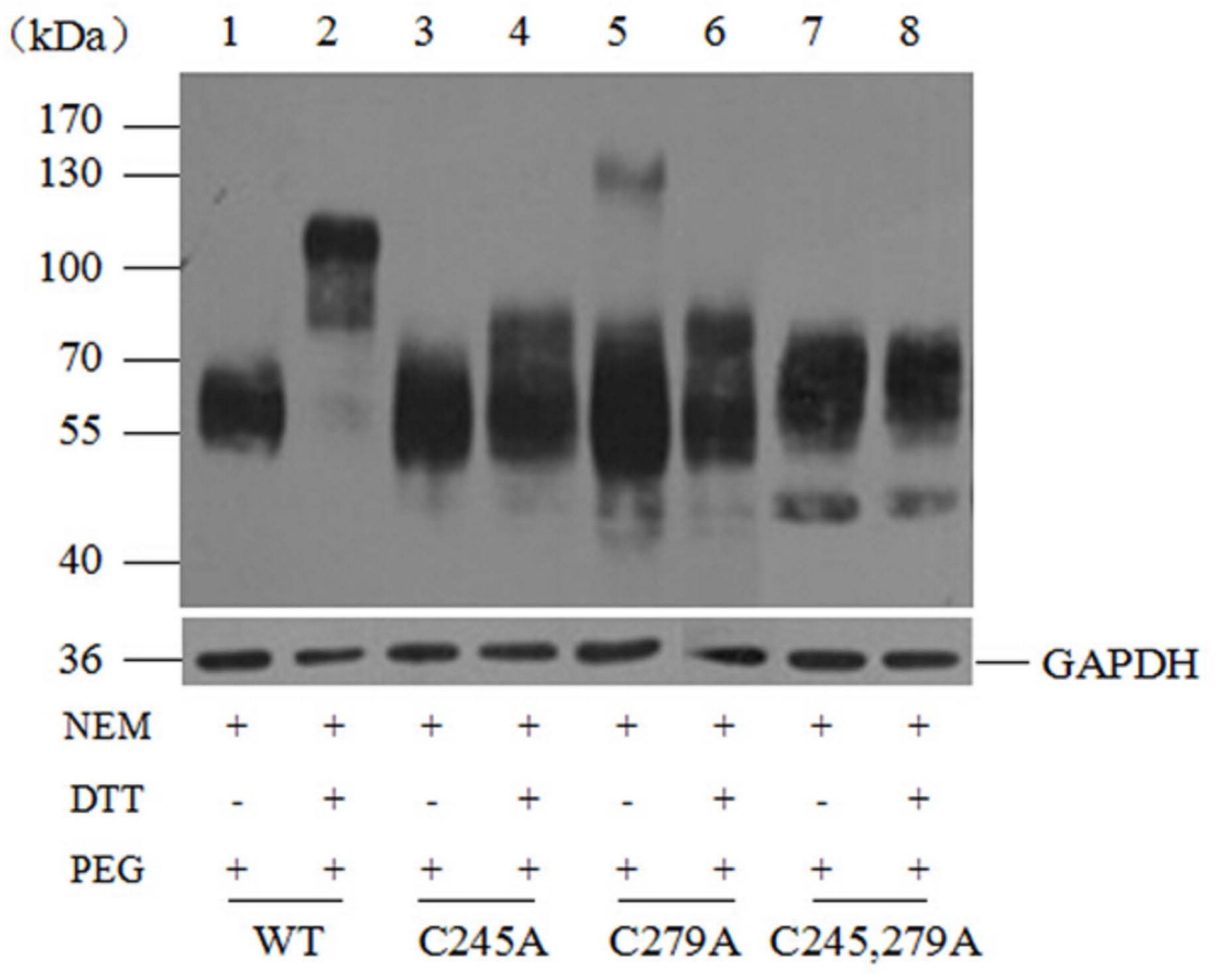
Western blotting analysis of SNAT2-HA wild-type and mutants C245A, C279A, C245,279A modified by NEM and mPEG-Mal. Lysates of HEK293T cells expressing either wild-type or mutant transporters, C245A, C279A, C245,279A were processed by sequential treatment of NEM, DTT, and mPEG-Mal under denatured condition. Controls were treated without DTT. The proteins were separated on an 8% SDS gel, detected by immunodetection with the mouse anti-HA tag antibody followed by the HRP-conjugated goat anti-mouse secondary antibody. Anti-GAPDH mouse monoclonal antibody was used to detect the GAPDH as an internal reference.

In contrast, for mutants C245A and C279A, the protein molecular mass without DTT treatment was 55~70 kDa (Fig 2, lane 7 and 9), while it was 55–80 kDa in the presence of DTT (Fig 2, lane 8 and 10). If only one instead of two cysteines can be modified by mPEG-Mal, due to the single mutation, a lower MW increase compared to SNAT2-HA WT is expected. Furthermore, the molecular mass of the C245,279A double mutant transporter, with or without DTT treatment, was 55~70 kDa (Fig 3, lane 7 and 8), equal to that of the wild-type in the absence of DTT. These results suggested the absence of disulfide bridges in this double mutant transporter, providing further evidence for a disulfide bridge between Cys245 and Cys279. Together, these results suggest that residues Cys245 and Cys279 are linked by a disulfide bond in the SNAT2 transporter protein.

### Expression and localization of wild-type and mutant SNAT2-HA

**Fig 4:**
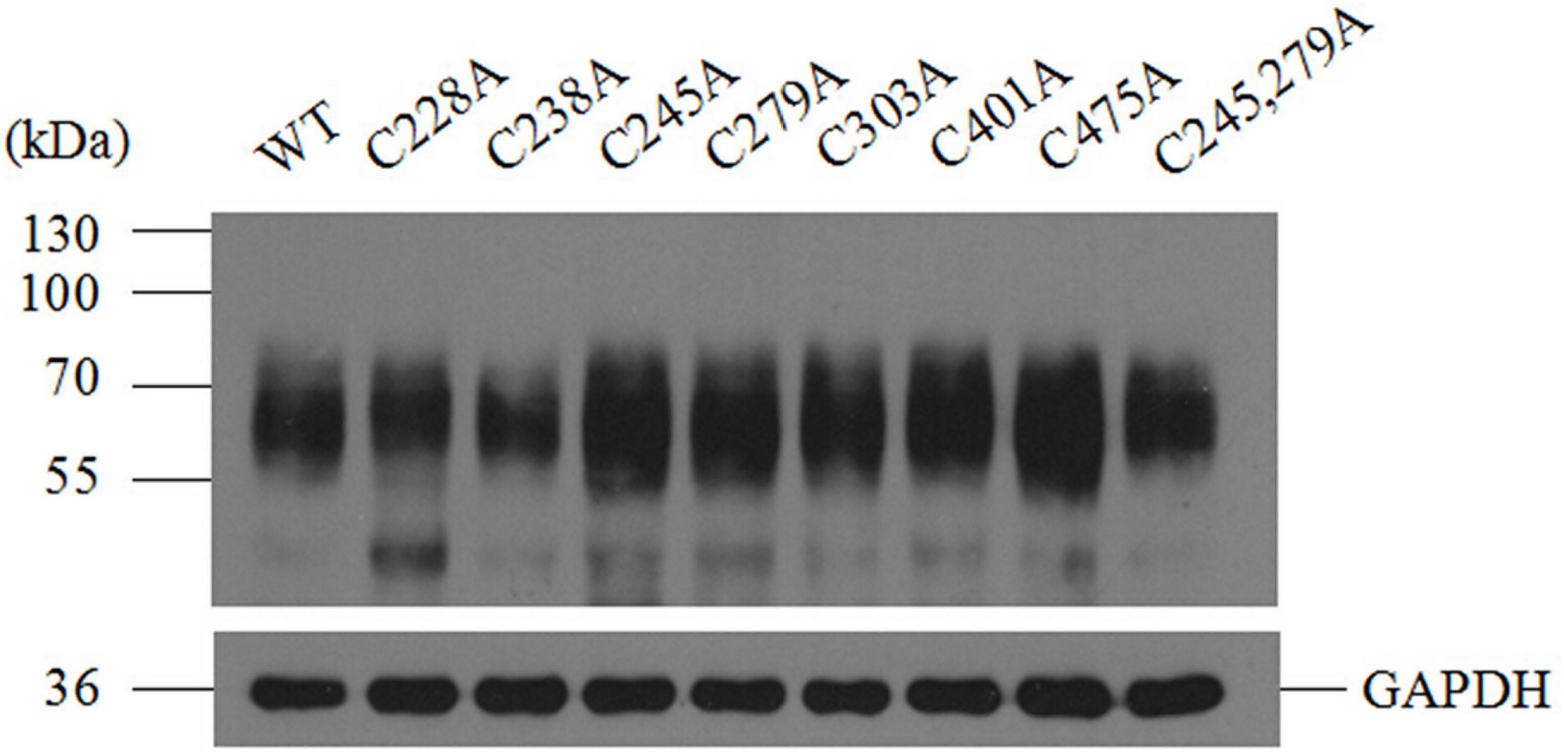
Western blotting analysis of SNAT2-HA wild-type and Cys to Ala mutants in cell total proteins. Lysates of HEK293T cells expressing either wild-type or mutant transporter were separated on an 8% SDS gel, detected by immunodetection with the mouse anti-HA tag antibody followed by the HRP-conjugated goat anti-mouse secondary antibody. Anti-GAPDH mouse monoclonal antibody was used to detect the GAPDH as an internal reference.

**Fig 5:**
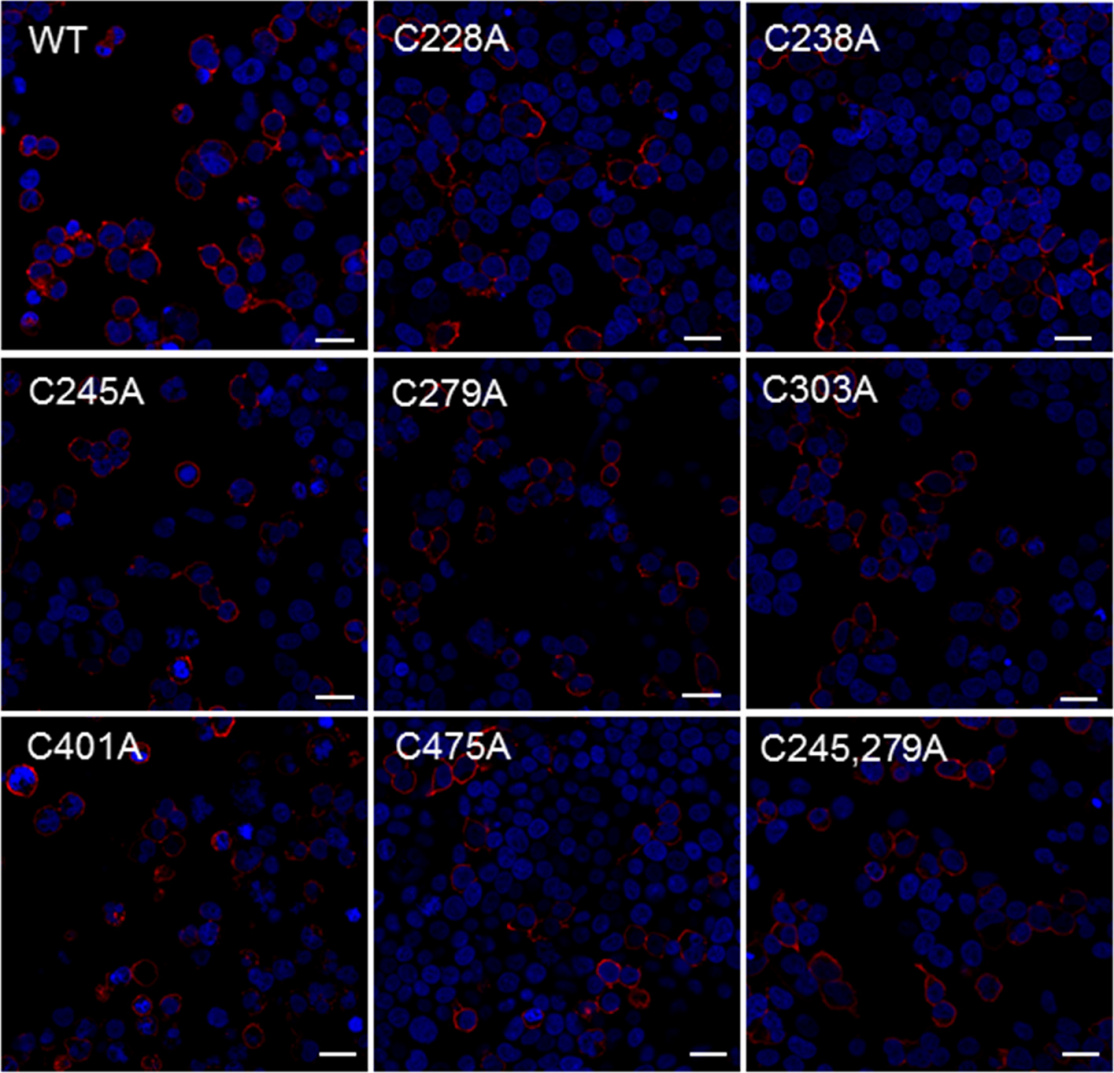
Immunofluorescence staining of SNAT2-HA wild-type and Cys to Ala mutants indicates correct membrane targeting of these transporters. HEK293T were transiently transfected with wild-type or mutant SNAT2-HA. 24 h after transfection, cells were incubated with the primary mouse anti-HA tag antibody followed by the secondary donkey anti-mouse Alexa Fluor 568 antibody. Nuclei were stained with DAPI. Scale bar = 25 μm.

To confirm cell expression of Cys to Ala mutants of SNAT2-HA, HEK293T cells were transiently transfected with plasmids encoding SNAT2-HA mutants and wild-type as a positive control. As can be seen in Fig 4, the molecular weight of all these mutant transporters were about 55~70 kDa, consistent with that of the wild-type protein. The images obtained from immunofluorenscence staining (Fig 5) showed all the SNAT2-HA mutants are localized at the surface of the cell, which means the disruption of the proposed disulfide bond does not affect trafficking of SNAT2 to the cell surface.

### Contribution of Cys residues and the disulfide bond to SNAT2 transport function

**Fig 6:**
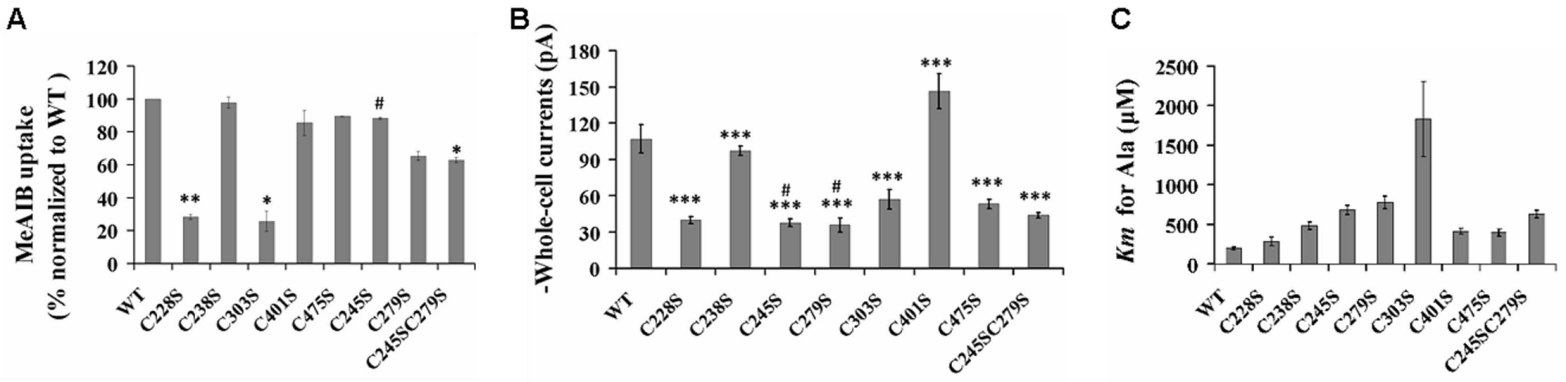
Transport function of Cys residues and the disulfide bond in SNAT2. A, MeAIB uptake of SNAT2 wild-type and Cys to Ser mutants. HEK293T were transiently transfected with wild-type or mutant SNAT2. Transport of [^14^C] MeAIB (40μM) was measured at 1 min in NaMes-containing buffer. The uptake of the vector was subtracted. B, average transport currents induced by the application of 10 mM alaine to SNAT2_WT_- and Cys to Ser mutants-expression cells. The currents of non-transfected cells were subtracted. C, the apparent affinities for the substrate L-alanine of SNAT2_WT_ and Cys to Ser mutant. *K*_*m*_, Michaelis constant. The black stars (*) indicate statistical significance compared with SNAT2_WT_ (***P < 0.001, * *P* < 0.05). The pound (#) indicates statistical significance compared with C245SC279S at the *P* <0.05 level. Values are means ± S.D. for at least three independent experiments.

The contribution of each Cys residue to SNAT2 transport activity was investigated by measuring MeAIB uptake experiments, transport currents induced by 10mM Ala and *K*_*m*_ for Ala, respectively. In order to reduce the effect of amino acid substitution on transport function of SNAT2, Cysteine residues at position 228, 238, 245, 279, 303, 401, and 475 were mutated to serine, respectively. The double mutation was generated by mutating both Cys245 and Cys279 to serine. Single mutant C228S or C303S reduced around 75% of the MeAIB uptake of SNAT2, demonstrated that both of C228 and C303 are important to transport function of SNAT2 (Fig 6A). Although C245S or C279S reduced the MeAIB uptake of SNAT2, the results were not significantly different compared with SNAT2_WT_. (The single mutant of C245S and C279S retained 88% and 65% of MeAIB uptake of SNAT2_WT_, respectively). The MeAIB uptake of double mutant of C245SC279S retained about 62% of that of SNAT2, almost was the same as C279S, appeared to be slightly reduced by the double mutation. Application of 10 mM alanine to SNAT2_WT_- and mutants-expression cells resulted in inwardly directed transport currents at 0 mV membrane potential (data not shown), average current of 106±12 pA for SNAT2_WT_. Except C405S, transport currents induced by 10 mM Ala of other mutants were decreased significantly compare to SNAT_WT_, but at least about 35% or above were retained (Fig 6B). C245S (39±3 pA) and C279S (37±6 pA) retained 37% and 35% of transport current compare to SNAT_WT_, respectively, while C245SC279S retained about 40% of transport current (44±2 pA), showed slight increased by double mutants. The apparent affinity of SNAT2_WT_ to alanien was 200±18 μM, C228S (289±56 μM, C238S (485±48 μM, C401S (415±37 μM) and C475S (401±49 μM) increased it 1~2 fold, showed that these four cysteines didn’t contribute too much to the alanine binding to SNAT2 (Fig 6C). While C303S increased the apparent affinity 8~9 fold (1833±475 μM), indicated C303 probably involved in binding Ala to SNAT2. The affinity ability to alanine of both C245S and C279S decreased 3~4 fold (*K*_*m*_ 689±60 μM for C245S and *K*_*m*_ 777±80 μM for C279) compared to SNAT2_WT_, respectively, double mutant’s *K*_*m*_ (634±44 μM) was the same as single mutants. All these results showed that the disulfide bridge formed between C245 and C279 is not absolutely required for the transport function of SNAT2, some Cysteines in SNAT2, such as C228 and C303, probably play an important role in the function of SNAT2 transport.

## Discussion

Disulfide bridges have been shown to play roles in various transmembrane proteins. However, the potential formation of a disulfide bridge in SNAT2 has never been investigated. In this study, we performed chemical modification with thiol-specific reagents, such as NEM and mPEG-Mal, providing the first evidence for disulfide bridging in SNAT2, and identifying C245 and C279 as candidates for forming the bridge.

mPEG-Mal is a membrane-impermeant reagent and has been shown to be a useful tool to study the topology of membrane proteins [43, 48–50]. This method has been used successfully to identify the disulfide bonds in transmembrane proteins, for example, acyl-coenzyme A:cholesterol acyltransferase 1 (ACAT1) [49] and the yeast vacuolar ATPase (V-ATPase) [50]. The double bond of maleimide in both NEM and mPEG-Mal can readily react with the thiol group of free cysteine, to form a stable carbon-sulfur bond [51]. NEM is commonly used as alkylating reagent of cysteine residues. Cys residues in putative transmembrane domains are inaccessible to NEM under native condition [52], while form covalent bonds with NEM under denatured condition [53]. The molecular weight of NEM is 125 Da, which renders modification of large proteins undetectable on SDS-PAGE. In contrast, mPEG-Mal has a large molecular mass of 5 kDa. For each thiol group, mPEG-Mal adds a PEG molecule, and the protein’s apparent molecular weight can be increased by more than 10–15 kDa, which can be easily detected by Western blotting analysis [48].

**Fig 7:**
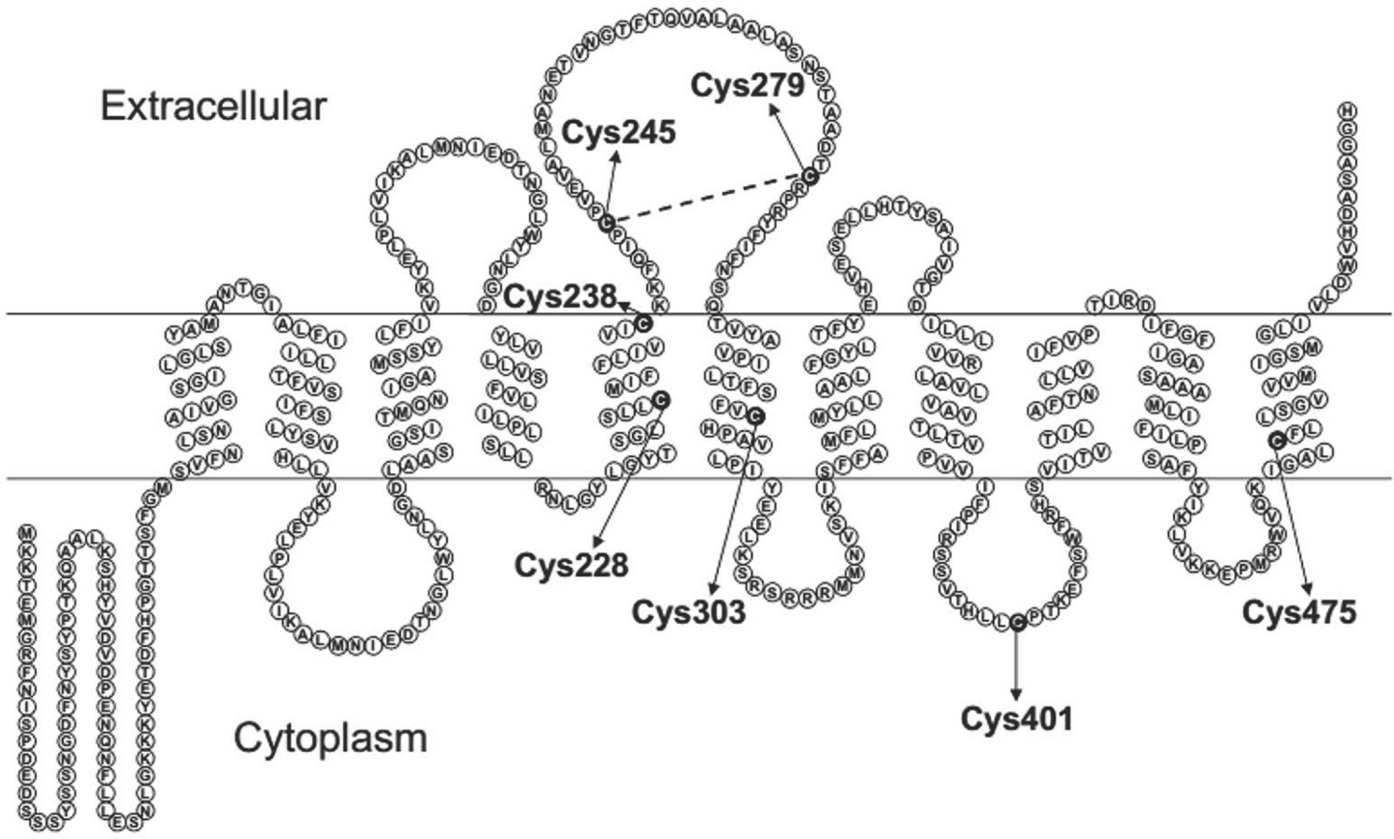
Topological model of SNAT2. The locations of 7 cysteine residues are indicated (arrows) in the proposed structure of SNAT2. The disulfide bond is shown as a dashed line.

In our present study, the wild-type and single Cys to Ala mutants C228A, C238A, C303A, C401A, and C475A of SNAT2-HA could be modified by two equivalents of PEG after DTT treatment, while C245A and C279A could bind only one PEG molecule per transporters unit. The most likely reason is that when one cysteine forming the disulfide bridge is mutated, the opposite cysteine may be blocked by some thiol-containing small molecules such as GSH, which made it couldn’t be modified by NEM. When the covalent compound was disrupted by DTT, the exposed thiol would be able to react with mPEG-Mal. In C245,279A double mutant, no PEG molecules could be attached after DTT treatment, which provided further evidence that Cys245 and Cys279 are linked by a disulfide bond. Together, these results support a disulfide bridge formed between Cys245 and Cys279 in the third extracellular loop, as shown in Fig 7.

There are 7 cysteine residues in SNAT2, four of which are highly conserved across the SLC38 family transporters. These residues are Cys228, Cys245, Cys279, and Cys303. As can be seen in the topology structure in Fig 7, Cys228 and Cys303 are respectively located in TM5 and TM6, while Cys245 and Cys279 are present on the third extracellular loop. Interestingly, a recent study showed that the conserved cysteine residues Cys249 and Cys321 in SNAT4 [47], which reside in the position corresponding to Cys245 and Cys279 of SNAT2, were linked by a disulfide bond. SNAT4 is another subtype of System A, which shares 57% sequence homology with SNAT2. The disulfide bond forming-cysteines of both SNAT2 and SNAT4 are present on the large extracellular loop, which may be a conserved structure symbol of SNAT family members. This has also been found in GABA and glutamate transporters [54, 55], and may suggest a general structural feature among these transporters of different families.

Homology models of the SNAT2 structure have been proposed [35, 56], based on the LeuT folds as a template. However, the structure of the extracellular loops cannot be modeled based on these structures. Therefore, the proposed disulfide linkage can provide important constraints for future modeling of the large extracellular loop between predicted TMs 5 and 6.

Disulfide bridges have been reported to play vital roles in the trafficking to the cell surface of protein. In CD36, the formation of disulfide bonds is required for protein transportation from the endoplasmic reticulum to Golgi [57]. A disulfide bond in the dopamine transporter is necessary for its delivery to the cell surface, while not essential for the uptake function. For the ATP-binding cassette (ABC) transporter ABCB6 and sulfonylurea receptor 1 (SUR1)/ABCC8, the breaking of disulfide bonds is closely related to protein degeneration [58]. Study of hPAT1 shows a dramatic effect on its transport function when the disulfide bond is disrupted [43]. Single cysteine residues are also found to be important for the structural integrity and transport function in proteins such as many GPCRs and some transporters [45, 46, 59–61]. However, we found that the disulfide bridge in SNAT2 is not essential for its trafficking to the plasma membrane (Fig 5), which is also observed in SNAT4 and hPAT1 [47, 60]. We also studied the effect of cysteines and the disulfide bridge on SNAT2 transport function by measuring MeAIB uptake, transport current at 10 mM alanine and apparent affinity to alanine (Fig 6). The highly conserved C228 and C303 are very important to transport function compared with SNAT2_WT_. The single mutant of C245S, C279S mutation did not significantly impair the transport activity of SNAT2_WT_. The double mutant transporter showed reduced MeAIB uptake, increased transport current at 10 mM alanine, and similar to apparent affinity, compare to single mutant of C245S, C279S mutation. but could still retain transport activity. This result indicates that the disulfide bond in SNAT2, while having some impact on transport, is not required for the transport function.

Recent studies have shown that SNAT2 does not only function as a transporter, but also function as a hybrid transporter-receptor (transceptor). As a member of system A transporters, SNAT2 carries out net transport of amino acids, therefore modulates the mTOR pathway through regulating intracellular amino acid concentration [32]. As a transceptor, SNAT2 can sense extracellular amino acid concentration and activate intracellular downstream signaling pathways such as mTOR, GCN, PI3K and CXCR4 pathways [35, 62–64]. The disulfide bridge in SNAT2 may play essential roles in signal transduction through thiol-disulfide exchange reactions, which remains to be shown [65].

In conclusion, a disulfide bridge between Cys245 and Cys279 in SNAT2 was identified in this paper, and our current results will be a foundation for the future study of SNAT2 and other members of SLC38 family, providing constraints for the structural fold for the important extracellular loop between TMs 5 and 6.
